# Quantifying heterogeneity in ecohydrological partitioning in urban green spaces through the integration of empirical and modelling approaches

**DOI:** 10.1007/s10661-023-11055-6

**Published:** 2023-03-15

**Authors:** Jamie Lee Stevenson, Christian Birkel, Jean-Christophe Comte, Doerthe Tetzlaff, Christian Marx, Aaron Neill, Marco Maneta, Jan Boll, Chris Soulsby

**Affiliations:** 1 Department of Geography, University of Aberdeen, Aberdeen, UK; 2 Department of Geography and Water and Global Change Observatory, University of Costa Rica, San José, Costa Rica; 3 School of Geosciences, University of Aberdeen, Aberdeen, UK; 4 IGB Leibniz Institute of Freshwater Ecology and Inland Fisheries, Berlin, Germany; 5 Geographisches Institut, Humboldt University Berlin, Berlin, Germany; 6 Northern Rivers Institute, University of Aberdeen, Aberdeen, UK; 7 Water Resources Management and Modelling of Hydrosystems, Technische Universität Berlin, Berlin, Germany; 8 Department of Geosciences, University of Montana, Missoula, USA; 9 Civil and Environmental Engineering, Washington State University, Pullman, WA USA; 10 Department of Ecohydrology, Leibniz Institute of Freshwater Ecology and Inland Fisheries, Berlin, Germany

**Keywords:** Urban ecosystem services, Water balances, Ecohydrological modelling, Precipitation partitioning, Infiltration, Evapotranspiration

## Abstract

Urban green spaces (UGS) can help mitigate hydrological impacts of urbanisation and climate change through precipitation infiltration, evapotranspiration and groundwater recharge. However, there is a need to understand how precipitation is partitioned by contrasting vegetation types in order to target UGS management for specific ecosystem services. We monitored, over one growing season, hydrometeorology, soil moisture, sapflux and isotopic variability of soil water under contrasting vegetation (evergreen shrub, evergreen conifer, grassland, larger and smaller deciduous trees), focussed around a 150-m transect of UGS in northern Scotland. We further used the data to develop a one-dimensional model, calibrated to soil moisture observations (KGE’s generally > 0.65), to estimate evapotranspiration and groundwater recharge. Our results evidenced clear inter-site differences, with grassland soils experiencing rapid drying at the start of summer, resulting in more fractionated soil water isotopes. Contrastingly, the larger deciduous site saw gradual drying, whilst deeper sandy upslope soils beneath the evergreen shrub drained rapidly. Soils beneath the denser canopied evergreen conifer were overall least responsive to precipitation. Modelled ecohydrological fluxes showed similar diversity, with median evapotranspiration estimates increasing in the order grassland (193 mm) < evergreen shrub (214 mm) < larger deciduous tree (224 mm) < evergreen conifer tree (265 mm). The evergreen shrub had similar estimated median transpiration totals as the larger deciduous tree (155 mm and 128 mm, respectively), though timing of water uptake was different. Median groundwater recharge was greatest beneath grassland (232 mm) and lowest beneath the evergreen conifer (128 mm). The study showed how integrating observational data and simple modelling can quantify heterogeneities in ecohydrological partitioning and help guide UGS management.

## Introduction

Urban green spaces (UGS) provide diverse and important ecosystem services (Derzken et al., 2017; Dickenson & Hobbs, 2017; Reyes-Riveros et al., 2021) and have the potential to act as an environmental buffer to the impacts of climate change and expanding urbanisation (Mathey et al., 2011; Sánchez et al., 2018). In a hydrological context, this buffering is manifested through the presence of pervious surfaces which can facilitate infiltration, soil storage and consequently groundwater recharge (Bai et al., 2018; Ellis, 2012; Yang & Lee, 2021). Further, evapotranspiration from vegetation returns water to the atmosphere (Mukherjee & Takara, 2018). These characteristics enhance urban ecosystem resilience against known hydrological impacts of climate change such as, for example, extreme precipitation (P) events and changes to groundwater recharge dynamics (Hughes et al., 2021), which can exacerbate the hydrological impacts of urbanisation such as increased flood or drought risk (Güneralp et al., 2015; Miller & Hutchins, 2017). Consequently, UGS are increasingly viewed as ‘green infrastructure’ (GI) in cities and towns and integrated into urban planning which aims to mitigate environmental impacts, improve urban sustainability and quality of life.

However, the specific characteristics of vegetation (e.g. species, height, density, location), in combination with the characteristics of the underlying soils, within UGS directly influences the exact nature of the ecohydrological partitioning of rainfall into ‘green’ water fluxes to the atmosphere (evaporation and transpiration) and ‘blue’ water fluxes (groundwater recharge and streamflow generation). Therefore, understanding similarities and differences in this partitioning is required to inform management of vegetation cover according to the required hydrological goal for the GI (e.g. greater groundwater recharge or higher levels of evapotranspiration). For example, there have been differences in transpiration amounts between individual conifer and deciduous species observed in Los Angeles (Pataki et al., 2011). Elsewhere in the USA, Peters et al. (2011) found grass to have higher rates of evapotranspiration in midsummer, per unit cover area, than evergreen conifers or deciduous broadleaf trees. Greater rates of soil infiltration have also resulted when urban trees are coupled with shrubs and grasses (Wang et al., 2018). Further, faster growing species with higher Leaf Area Index (LAI) values may provide greater urban cooling through higher evapotranspiration (Rahman et al., 2015). Kuhlemann et al. (2021) also found higher levels of interception and transpiration from urban trees as opposed to shrub and grassland species, which resulted in slower replenishment of soil water under the former following drought. More widely, Kim and Jackson (2012) identified how vegetation type exerts a strong control on groundwater recharge globally, though results varied across climate and soil variables, meaning further research is required to more fully elucidate the role of vegetation on groundwater recharge. Clearly, therefore, the particular effects of UGS on water partitioning are highly variable and strongly dependent on the local geography (e.g. climate, soils, native species abundance, management). However, process-based studies in urban ecohydrology are less common than non-urban environments and the evidence base is sparse, with more empirical studies required to create a diverse evidence base and show the geographic variability in the impact of GI (Marchionni et al., 2019). Further, in recent years, the use of stable water isotopes to enhance process-based understanding of urban hydrology has been stressed (c.f. Ehlerringer et al., 2015), with studies which have incorporated these tracers gaining informative insights into ecohydrological partitioning (e.g. Kuhlemann et al., 2021; Marx et al., 2022; Wang et al., 2016).

Where available, empirical UGS datasets have usefully been coupled with ecohydrological modelling approaches to quantify differences under contrasting vegetation types. For example, Gillefalk et al. (2021) used an advanced tracer-aided model to quantify the impact of vegetation type on water partitioning in central Berlin, finding that green water fluxes were greatest from trees, followed by grass and then shrubs; mainly due to higher interception losses from trees. The authors also identified the importance of vegetation type on the regulation of groundwater recharge, given water ages were higher in the soil water below trees than the other vegetation types, leading to lower and older groundwater recharge fluxes. Furthermore, Meili et al. (2020) developed an urban-specific, fully coupled energy and water balance model, demonstrating that fully grass covered ground and higher values of LAI increased relative humidity, which would feed back into evapotranspiration rates. Though very informative, such models can require extensive or diverse input datasets, be highly parameterised (risking over-parametrisation whereby not all parameters may be inferred from the data and thus increase the risk of overfitting), and/ or have high computational demands. All such factors can then limit the models’ transferability to relatively rapid, multi-site ecohydrological assessment in urban settings. Given this, there is a requirement for simpler, more transferable, ecohydrological models that can be applied to differing vegetation types within an urban setting to estimate differences in ecohydrological partitioning, in particular transpiration, evaporation and groundwater recharge. Moreover, such model development needs to be data driven, with ecohydrological monitoring undertaken in previously unstudied locations to extend knowledge on how urban vegetation type impacts water partitioning and thus derived ecosystem services such as urban heat island effect mitigation and groundwater recharge.

To address this research need, we aimed to characterise the heterogeneities in ecohydrological partitioning between five broad vegetation types (grassland, evergreen shrub, evergreen conifer, smaller deciduous tree and larger deciduous tree) in an UGS in Aberdeen, northern Scotland. We employed a data-driven approach through the collection of hydroclimatic, soil moisture, soil isotope, precipitation isotope, sapflux and time-lapse geophysical data over the course of 1 year’s growing season. These data were then further used to develop a new, low parameter, one-dimensional conceptual ecohydrological model to resolve and estimate ecohydrological fluxes such as evaporation, transpiration and groundwater recharge under contrasting vegetation types. In doing so, we addressed the following specific research objectives:

1. To characterise temporal dynamics of key water balance components that reflect ecohydrological partitioning within an established UGS.
2. To identify dominant controls on the observed dynamics of this ecohydrological partitioning under contrasting vegetation types.
3. To apply insights from 1 and 2 to develop and apply a one-dimensional, relatively simple, ecohydrological model to estimate flux partitioning under contrasting vegetation types.

## Study site

The study was undertaken in the Cruickshank Botanical Garden (CBG) of the University of Aberdeen in the expanding city of Aberdeen, NE Scotland, which contains native, non-native and ornamental plant species of varying sizes and ages (see University of Aberdeen, 2022). Typical of established UGS, the site has had multiple uses in the past, resulting in intermittently disturbed upper soils. Geologically, the bedrock in the area is composed of metamorphic psammite and semipelite, in contact with the Aberdeen granite to the West and conglomerates and sandstones to the East. The bedrock is overlain by glacial till, sands and gravel deposits (Edina Digimap, 2022). Soils are ~ 2 m deep and are typically mineral podzols and brown soils. The climate of Aberdeen is temperate/boreal oceanic with average precipitation of ~ 850 mm per year, with most rainfall events being of low intensity and generally spread fairly evenly throughout the year (average monthly standard deviation of ~ 18 mm), whilst snow accounts for < 5% of annual precipitation (Meteostat.net, 2022). Monthly mean temperatures range from ~ 3 °C in January to ~ 14 °C in July and August, with rises and falls between these months relatively gradual and consistent (Meteostat.net, 2022).

**Fig. 1:**
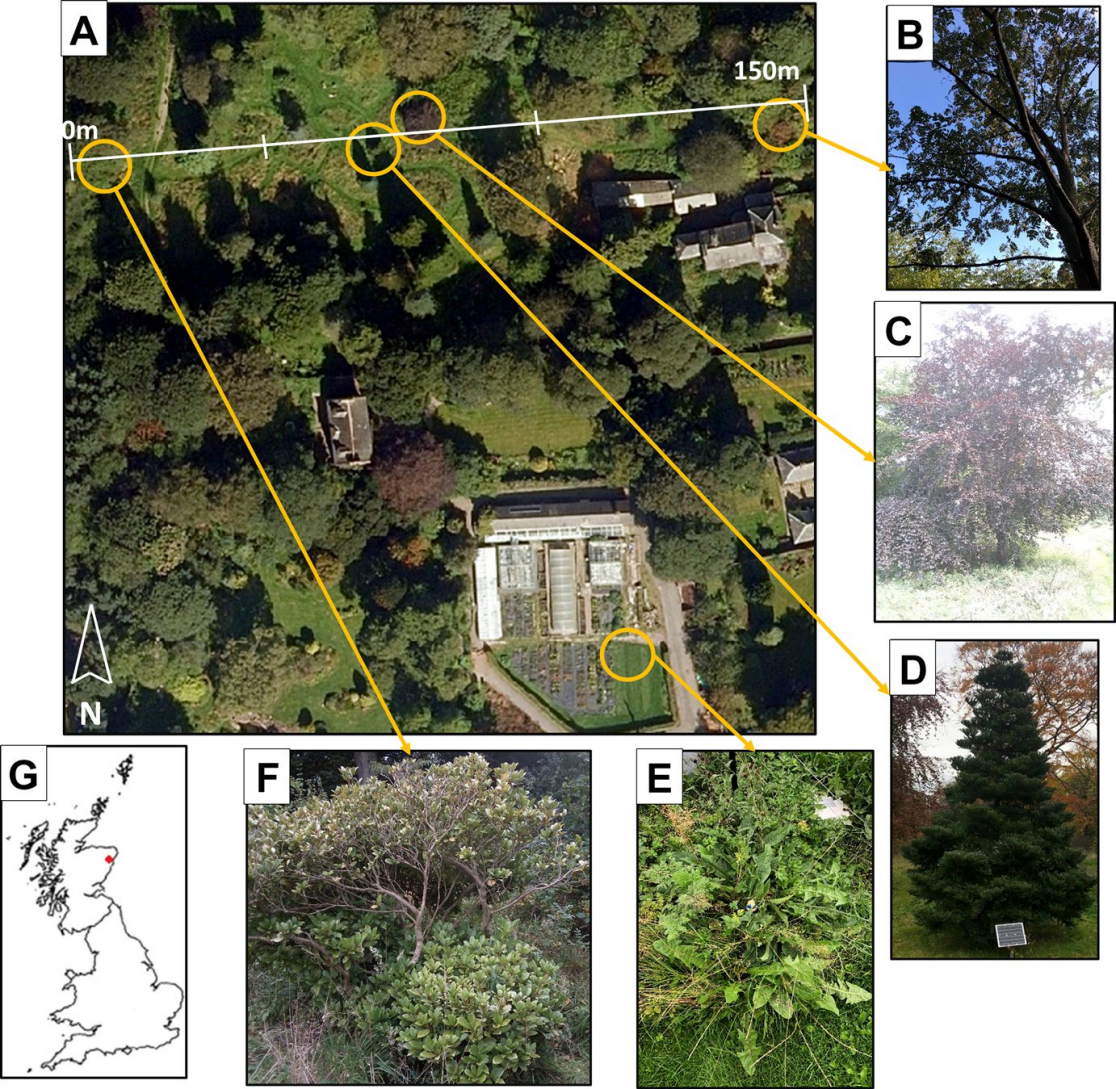
Overview of the study site (**A**) with locations of the smaller deciduous tree (**B**), larger deciduous tree (**C**), evergreen conifer (**D**), grassland (**E**) and evergreen shrub (**F**) circled. Wider geographical reference displayed in **G**. The weather station and precipitation collector were installed at the same location as the grassland (**E**)

Ecohydrological monitoring was focussed around a 150-m transect of the CBG (Fig. 1). The area contained contrasting vegetation types to ensure close proximity of monitoring sites to minimise local variation in hydroclimatic variables which could influence observed ecohydrological dynamics. The plot was also a less intensively managed area of the CBG with the least amount of soil disturbance.

## Methods

### Vegetation plots

Five unirrigated vegetation types were selected for intercomparison (Fig. 1), consisting of a evergreen shrub (*Skimmia japonica*; ~ 15 years old, ~ 1.8 m tall), an evergreen conifer (*Abies korena*; ~ 30 years old, ~ 6 m tall), larger deciduous tree (*Fagus sylvatica*; ~ 60 years old, ~ 10 m tall), smaller deciduous tree (*Sorbus glabrescens*; ~ 20 years old, ~ 6 m tall) and grassland site, which contained a variety of species associated with this habitat, such as *Taraxacum* spp., to a height of ~ 0.4 m*.* When selecting these generic vegetation types, we aimed to keep them both broad and diverse in terms of physiology, age, size and being native or non-native in an effort to ensure results were transferable to other UGS sites. Furthermore, it was hypothesised that diverse vegetation types may display contrasting ecohydrological dynamics and partitioning, thereby providing results that could inform practical management decisions where specific ecosystem services may be targeted.

Four of the vegetation types (evergreen shrub, evergreen conifer, larger and smaller deciduous trees) were located on a 150-m transect, with the grassland c.150 m to the south to keep the plots as close together as practically possible (Fig. 1). Furthermore, this facilitated a geophysical transect to be surveyed to spatially connect four of the individual plot findings and place them in context of the wider CBG. The transect ranged from an elevation of ~ 28 m above sea level (m.a.s.l) at the start to ~ 18 m m.a.s.l at the end. The steepest part of this decline occurred in the first 30 m, decreasing by ~ 6.5 m, before experiencing a more consistent, gentle, decline. The evergreen conifer, larger deciduous and grassland sites had similar soil properties being an undifferentiated silty-clay-loam subsoil, with distinct organic-rich surface horizon. The evergreen shrub site (and immediate surrounding area) located upslope contained a greater proportion of sandy material, whilst the downslope area encompassing the smaller deciduous tree evidenced greater soil disturbance and had an increased silt component in the upper-most soils.

### Hydroclimatic data

An Environmental Measurements Ltd weather station fitted with a Campbell Scientific CR800 logger was installed and operated from January 2021 to November 2021 to record precipitation, air temperature, windspeed, net radiation, barometric pressure and relative humidity at 15-min intervals, which were then aggregated to daily values. Unfortunately, the relative humidity sensor failed, with hourly data instead sourced from the nearby (~ 3 miles) Met Office-operated weather station at Dyce (Meteostat.net, 2022). The relatively close proximity, and high correlation with other meteorological variables measured at both sites, underlined the suitability of the Dyce derived data. The location of the weather station (Fig. 1) was chosen to ensure the largest and most obvious equipment was within a more secure area of the CBG, whilst still being representative of, and in close proximity to, the main transect. Potential evapotranspiration (PET) was calculated from the Penman–Monteith equation using the *ET.PenmanMonteith* function from the Evapotranspiration R package (Guo et al., 2016). The short crop version of the equation was applied to the grassland site, whilst the long crop version was applied to the other sites to account for the contrast in vegetation height.

### Soil moisture and geophysical data

Between December 2020 and November 2021 Odyssey Xtreem multi-profile soil moisture capacitance probes were installed and operated directly under each vegetation type. These recorded soil moisture values every 15 min at 10, 20, 40, 60 and 100 cm from the surface, with values aggregated to daily averages. To allow time for the minimal soil disturbance from installation to subside, data prior to January 2021 was discarded. In setting up the study site, we were aware that individual sensors under contrasting vegetation types would be unlikely to capture the full heterogeneity of ecohydrological partitioning by vegetation. Thus, geophysical techniques were used to link sensor observations to more spatially extensive patterns of soil moisture variability.

Consequently, three geophysical surveys (electrical resistivity tomography, ERT) were undertaken along the transect delineated in Fig. 1 in October 2020, July 2021 and November 2021. These provided insight into the structure and characteristics of the subsurface and indicated larger-scale spatial patterns and temporal changes in soil moisture across the seasons. The ERT set up used 72 electrodes at 2.5 m spacing with data collected using an IRIS Syscal Pro resistivimeter employing dipole–dipole and multigradient arrays, which were subsequently jointly inverted for each survey date using Res2Dinv (version 3.59), incorporating the transect topography (following the workflow by Comte et al., 2012). The three surveys were timed so as to capture conditions at the start and end of the period of interest and at a point between when soils were expected to be at their driest; therefore capturing the most extreme changes in the annual drying and re-wetting cycle.

### Sapflux data

ICT International SFM1 sap flow metres were installed in the evergreen shrub, evergreen conifer, larger deciduous and smaller deciduous trees; it was impractical to make similar measurements in the grassland vegetation. The sensors use the Heat Ratio Method (see Burgess et al., 2001 for details) to infer sapflux rates at 15-min intervals which were then aggregated to daily totals. To convert raw data into volumetric values can be difficult given the number of parameters needed, some of which required further invasion into the plants which was not deemed suitable in the CBG. Therefore, the data was normalised (via the standard ‘min–max’ normalisation approach) to show the general dynamics of sapflux as a proxy for transpiration.

### Isotope data

#### Precipitation

Daily P samples for isotope analysis were collected at the weather station location (Fig. 1) using an adapted ISCO 3700 streamflow autosampler. Paraffin was added to bottles prior to deployment to prevent fractionation. All samples were analysed for deuterium (δ^2^H) and oxygen-18 (δ^18^O) isotopes at the University of Aberdeen using a Los Gatos TIWA-45-EP laser isotope analyser (precision of ± 0.2‰ for δ^2^H and ± 0.05‰ for δ^18^O).

#### Soil water

Soil samples were taken with a soil auger for isotope analysis of soil water from under each vegetation type at 0–10 cm, 11–20 cm and 21–35 cm depths, at monthly intervals between April and September. Three replicate samples were taken for each depth and month at each vegetation plot to capture heterogeneities in soil water isotope signals. As per Kuhlemann et al. (2021), samples were placed, avoiding air inclusion, in WEBAbags (Silver Range, Weber Packaging, Germany), immediately heat sealed and shipped using a direct courier for next day delivery to the Leibniz Institute of Freshwater Ecology and Inland Fisheries, Germany, IGB. Samples were then analysed for δ^2^H and δ^18^O by first being inflated with dry air, equipped with a silicon septum and stored for 48 h to equilibrate. A needle attached to a tube was then inserted into the bags via the septum to analyse the vapour phase using a Los Gatos TIWA-45-EP isotope analyser (precision of ± 0.2‰ for δ^2^H and ± 0.05‰ for δ^18^O). For correction, nine 10 mL standard water samples of δ^2^H and δ^18^O (spanning a range which generously encompassed the expected soil water isotope signal range) were used during each measuring routine.

#### Additional ecohydrological data

We calculated LAI timeseries from field observations, sapflux data and literature, resulting in the evergreen shrub and evergreen conifer having time in-variant LAI values of 6 m^2^/m^2^ and 7 m^2^/m^2^, respectively. For the time-variant canopies, a trapezoidal shape was employed, in the absence of repeated LAI measurements.

The timing of rises, peaks and decreases of this trapezoid were directly guided by field observations and sapflux measurements where available, resulting in an initial value on 1st March of 1.5 m^2^/m^2^ for the larger deciduous tree which remained constant before rising to 6 m^2^/m^2^ between 12th May and 17th July, and then reducing to 3.2 m^2^/m^2^ between 01^st^ October and the end of the modelling period (31st October). The grassland timeseries followed the same temporal pattern but rose from 1.5 to 4 m^2^/m^2^, ending the modelling period with a value of 2.49 m^2^/m^2^.

### One-dimensional ecohydrological model development

#### Model structure

**Fig. 2:**
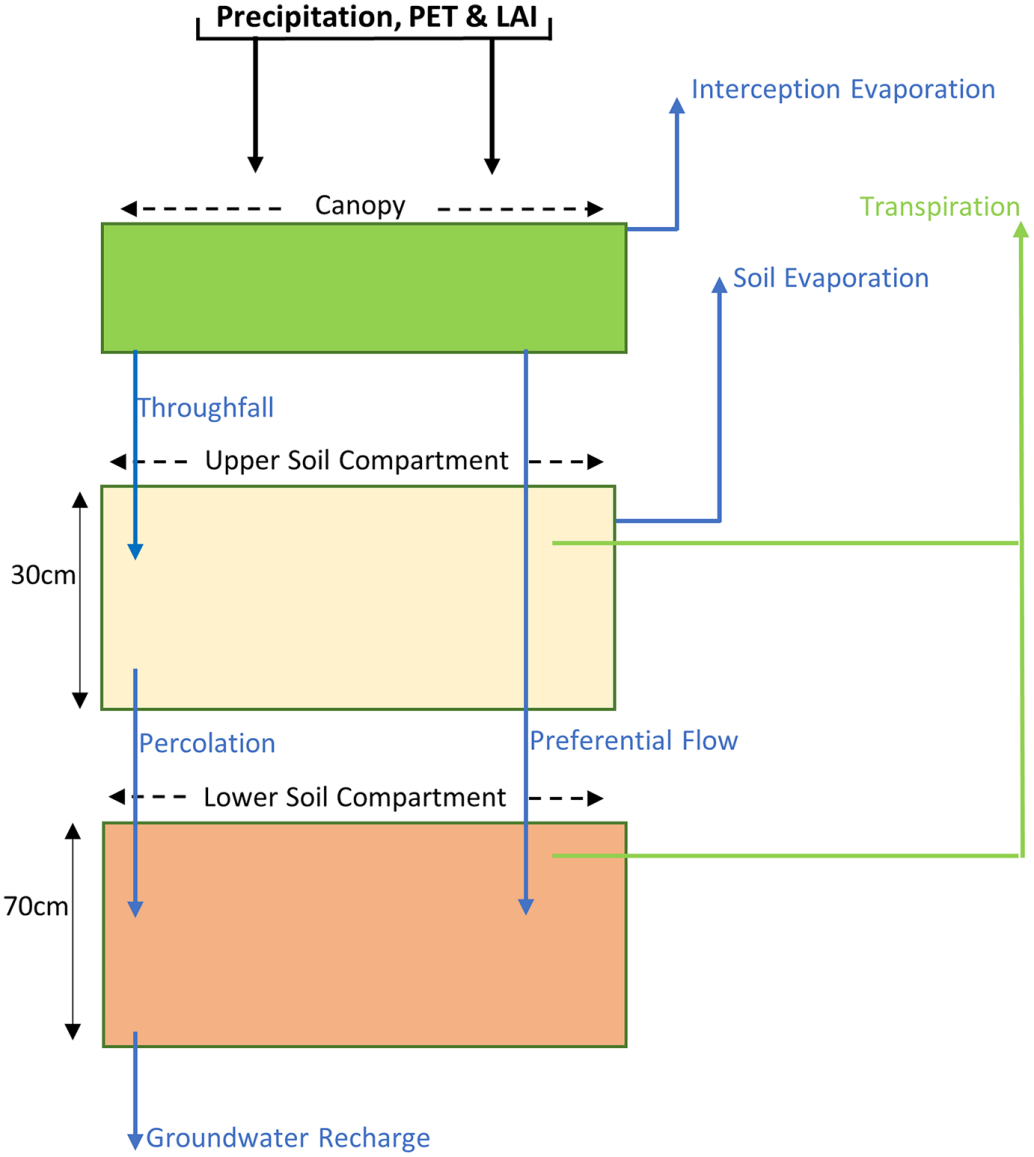
Schematic diagram of the one-dimensional plot-scale ecohydrological model (EcoHydroPlot). See ‘Model structure’ for full detail, parameters and governing equations

We developed a new one-dimensional, nine-parameter, plot-scale conceptual ecohydrological model (EcoHydroPlot) in the R coding language (R Core Team, 2022). Figure 2 shows the basic structure of the model which is driven by inputs of P, PET and LAI. EcoHydroPlot was run on a daily timestep as this was deemed sufficient to capture changes in soil moisture storages (even with the occurrence of sporadic higher-intensity events given that overland flow was not observed during these events), as well as the green and blue fluxes in a relatively low energy environment. The model was developed and applied to all sites apart from the *Sorbus glabrescens* as equipment installation showed the highly disturbed nature of the soil at this site (which impacted soil moisture dynamics) mitigated against its use for developing a simple, transferable, model (see ‘Vegetation plots’ and ‘Soil moisture’ sections for further detail).

In the modelling process, LAI was first used to calculate the Surface Cover Fraction (SCF) of the canopy using Beers Law (Eq. 1), which required the calibration of a radiation extinction parameter (*rE*). The SCF and a calibrated interception threshold parameter (*α*) then calculated the depth of water a dry canopy could intercept (Int_Max_; Eq. 2, Šimůnek et al., 2013) at each timestep (i). Any depth of P in excess of Int_Max_ was routed to the upper soil compartment as throughfall (*T*_h_). Additional *T*_h_ was generated by any depth of Int_Max_ that caused the running canopy water balance to exceed a canopy saturation volume (C_Sat_) which was estimated at each timestep by using the LAI within a relationship developed through literature (c.f. Gillefalk et al., 2021; Kuhlemann et al., 2021) and assessing modelled output (Eq. 3).

1 $$SCF\left[i\right]= 1-\mathrm{exp}(rE*LAI\left[i\right])$$

2 $$In{t}_{Max}[i]=\left(\alpha *LAI\left[i\right]\right)*(1-(1 / (1+\left(\frac{SCF\left[i\right]*P\left[i\right]}{\alpha *LAI\left[i\right]} \right))))$$

3 $${C}_{sat}[i]= 0.2001+(LAI\left[i\right]*0.3001)$$

If *T*_h_ exceeded 3 mm in the evergreen shrub, evergreen conifer and larger deciduous site, preferential flow (*P*_F_) was initiated whereby a calibrated parameter (*PF*_*Scale*_) controlled the proportion of *T*_h_ which was routed directly into the lower soil compartment. This threshold was refined based on preliminary model results from applications to the different vegetation types. In the case of the grassland site, *P*_F_ was initiated if the addition of *T*_h_ into the upper soil compartment caused the simulated store to exceed the calibrated *S*_max_ parameter, which represented the theoretical maximum soil water capacity, as this yielded more representative results. No overland flow component was included as field observations demonstrated that this did not occur during the study period.

Following Šimůnek et al. (2013), and consistent with the ethos of creating a low parameter model, SCF was also used to separate PET into potential transpiration (*T*_p_) and potential evaporation (*E*_p_) via Eqs. 4 and 5, respectively. *E*_p_ was then taken entirely, if possible, from the interception store (Int_E_). Transpiration volume was then taken from the upper soil compartment (Tr_Upper_; Eq. 6), scaled according to simulated storage at each timestep (STO) using the calibrated *S*_max_ parameter. At this point, any remaining *E*_p_ volume was taken from the upper soil store (Soil_E_; Eq. 7).

4 $${T}_{p}\left[i\right]=SCF\left[i\right]*PET[i]$$

5 $${E}_{p}\left[i\right]=(1-SCF\left[i\right])*PET[i]$$

6 $${Tr}_{Upper}[i]= {T}_{p}\left[i\right]* \left(\frac{STO\left[i\right]}{{S}_{max}}\right)$$

7 $${Soil}_{E}[i]=(Ep\left[i\right]-In{t}_{E}[i])* \left(\frac{STO\left[i\right]}{{S}_{max}}\right)$$

Next, the percolation flux (*P*_c_) between the upper and lower soil compartments was calculated, controlled by the calibrated *S*_max_ parameter, the calibrated linear parameter *ks1* and the calibrated non-linear parameter *g1* (Eq. 8).

8 $${P}_{c}[i]=ks1*{\left(\frac{STO\left[i\right]}{{S}_{max}}\right)}^{g1}$$

After the addition of *P*_c_ to the lower soil store, and if *T*_p_ was still present, transpiration was allowed to be drawn from the lower compartment (Tr_Lower_, Eq. 9); scaled as per Tr_Upper_ though using the lower box storage at that timestep (GW) and the calibrated *GW*_max_ parameter, which acted in the same way as *S*_max_. Tr_Lower_ was not permitted in the grassland application given rooting depths were not observed to routinely exceed 30 cm. Finally, water could exit the lower compartment via groundwater recharge (*G*_r_; Eq. 10) using the same non-linear approach of *P*_c_.

9 $${Tr}_{Lower}\left[i\right]= {(T}_{p}\left[i\right]- {Tr}_{Upper}[i])* \left(\frac{GW\left[i\right]}{{GW}_{max}}\right)$$

10 $${G}_{r}[i]=ks2*{\left(\frac{GW\left[i\right]}{{GW}_{max}}\right)}^{g2}$$

#### Model calibration

For each model run, 100,000 unique parameter sets were generated using Latin-Hypercube sampling of the parameter space (LatinHyper function from the FME package; Soetaert & Petzoldt, 2010). The performance of each parameter set was assessed against observed values through the calculation of the modified Kling-Gupta KGE efficiency statistic (Kling et al., 2012) for simulations in both the upper and lower soil compartments. The KGE metric was chosen because of its consideration of variability, correlation and bias. Observed soil moisture point values within the soil column were aggregated based on the assumption that the 10, 20, 40, 60 and 100 cm soil moisture measurements could be assumed as broadly representative of the depths between 0 to 15 cm, 16 to 30 cm, 31 to 50 cm, 51 to 80 cm and 81 to 100 cm, respectively. Measurements at 10 cm and 20 cm were aggregated for use in the upper box and the remaining values aggregated for the lower box at each timestep. Only parameter sets with KGE values within the top tenth percentile of all KGE values for both compartments were retained. This approach was used to ensure optimum simulations only were selected, whilst, in the absence of formal uncertainty analysis, providing confidence intervals around average simulated values (median values reported in-text unless stated otherwise). For vegetation types where sapflux data (normalised according to data collection protocol) were available, an R^2^ statistic against normalised simulated transpiration values was also calculated to assess for statistically significant relationships between observed sapflux dynamics and the simulated transpiration dynamics. The results of these were not used as a formal calibration target as per the soil moisture, but rather as an independent model performance check.

For each model, application initial parameter ranges (guided by literature and site knowledge) were constrained on the basis of performance criteria detailed above, until further refinement resulted in worsening model performance. Moreover, each model output was checked after each run to ensure increases in formal performance criteria (KGE) were not at the cost of unrealistic simulations in other areas of the model. After each vegetation type had been refined, the global maximum and minimum (e.g. for inter-vegetation type) was identified for each parameter (Table 1). This range was then used to generate the initial 100,000 parameter sets for application to all vegetation types. Selection criteria, as above, was then used to retain behavioural parameter sets from this 100,000 for the individual model applications. Doing so ensured that each vegetation type would be included in what the model could reasonably represent, allowing results to be directly comparable and based on the model calibration process alone.

**Table 1:** Parameter calibration values

	**rE** **(-)**	**Alpha (mm)**	***S***_**max**_** (mm)**	**ks1** **(per day)**	**ks2** **(per day)**	**GW**_**max**_** (mm)**	**g1** **(-)**	**g2** **(-)**	**PF**_**Scale**_ **(-)**
**Inter-vegetation type initial parameter ranges**									
*Minimum*	− 0.463	0.5	100	0.5	2	300	2	1	0.37
*Maximum*	− 0.2	3.5	340	5	9	420	5	5	0.50
**Post-calibration retained median values (10th percentile, 90th percentile)**									
*Evergreen Shrub*	− 0.33 (− 0.43, − 0.23)	2.02 (0.83,3.17)	112 (102,140)	3.24 (1.48,4.47)	6.40 (3.80,8.48)	350 (309,401)	3.03 (2.17,4.30)	1.48 (1.14,1.83)	0.44 (0.39,0.49)
*Evergreen Conifer*	− 0.33 (− 0.44, − 0.23)	2.05 (0.84,3.20)	175 (148,228)	2.46 (0.86,4.36)	4.23 (2.37,7.39)	385 (340,413)	3.06 (2.17,4.52)	4.25 (3.19,4.85)	0.44 (0.38,0.49)
*Larger Deciduous*	− 0.33 (− 0.44, − 0.22)	2.06 (0.83,3.20)	268 (230,318)	3.28 (1.48,4.65)	4.01 (2.38,6.71)	365 (315,411)	3.54 (2.48,4.74)	2.30 (1.62,2.91)	0.47 (0.43,0.49)
*Grass*	− 0.33 (− 0.44, − 0.23)	2.04 (0.81,3.24)	150 (123,174)	1.87 (0.74,4.21)	5.49 (2.83,8.18)	355 (313,403)	3.99 (2.79,4.81)	3.71 (2.54,4.67)	0.44 (0.38,0.49)

## Results

### Hydroclimatic dynamics

**Fig. 3:**
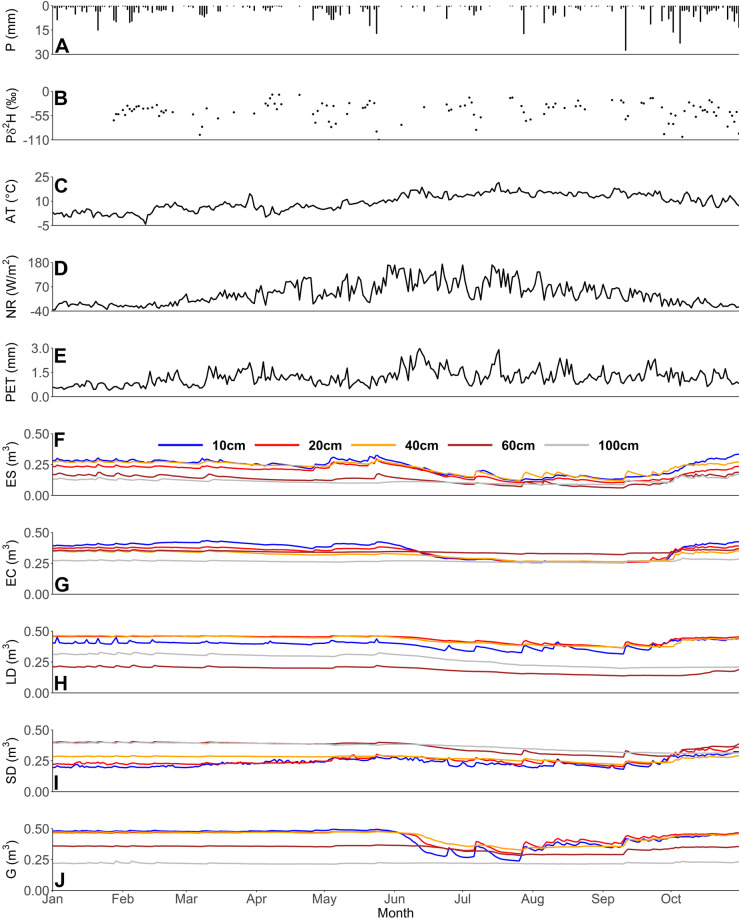
Timeseries of precipitation (**A**), deuterium isotopes of precipitation (**B**), air temperature (**C**), net radiation (**D**), Penman–Monteith long crop equation-derived PET estimates (**E**) and multi-depth soil volumetric water contents beneath the evergreen shrub (**F**), evergreen conifer (**G**), larger deciduous (**H**), smaller deciduous (**I**) and grassland (**J**) sites. Key to multi-depth colouration located in panel **F**, with in-print readers directed to the online version. Figure created in RStudio

Total P for the period 01/01/21 to 31/10/2021 was 575 mm; ~ 120 mm lower than the previous 10-year average as calculated from the nearby Met Office-operated Dyce weather station data (Meteostat.net, 2022). In particular, March, April, June, July and August were drier, though May and October were somewhat wetter. Air temperature followed expected dynamics, being at its lowest in January and the start of February, peaking in June and July (Fig. 3). March was around 1.3 °C warmer than the 10-year average of 5.5 °C, whilst April and May were notably cooler than their 10-year average being 5.3 and 8.3 °C, compared to long-term averages of 7.3 and 10 °C, respectively. The end of April and beginning of May saw a dip in the general rising trend of net radiation (Fig. 3), with this reducing PET (Fig. 3) before peaks in mid-June and mid-July. Furthermore, given higher temperatures were sustained until early October, PET also had relative peaks during late September.

### Soil water dynamics

#### Soil moisture

Temporal dynamics in soil moisture were similar under the different vegetation types, with soils relatively stable in their volumetric water content (VWC) until early June (Fig. 3). A rapid drying trend followed as a result of low frequency, low magnitude P events and increase in PET (Fig. 3), with soils, generally, experiencing a pronounced re-wetting from mid to late September as autumn rainfall commenced. As expected, upper soils were most dynamic in response to P inputs, whilst the lowest soils at 100 cm showed more attenuated responses (Fig. 3). There were, however, substantial differences in detail between the plots. Considering the soil column as a whole, the grassland had the greatest mean storage volume across the sites, whilst the sandier soil at the evergreen shrub site was by far the driest (Table 2). Furthermore, during general drying in early June, the grassland site upper soils experienced the most rapid reduction in VWC, then showing the most pronounced dynamics in response to P inputs during the subsequent dry period (Fig. 3). Contrastingly, during this time period, the two deciduous sites saw a more gradual reduction in VWC with relatively damped responses to P, whilst the evergreen conifer acted fundamentally differently with a pronounced drying trend and being much less responsive to P events (Fig. 3).

**Table 2:** Summary statistics covering 01/01/21 to 31/10/21 of soil moisture volumetric water content (VMC; m^3^ of water per m^3^ of soil)

	**Evergreen shrub**	**Evergreen conifer e**	**Larger deciduous**	**Smaller deciduous**	**Grassland**
10 cm Mean	0.23	0.35	0.39	0.23	0.43
10 cm SD	0.06	0.07	0.03	0.03	0.07
20 cm Mean	0.19	0.34	0.44	0.25	0.43
20 cm SD	0.05	0.05	0.03	0.03	0.05
40 cm Mean	0.22	0.31	0.43	0.27	0.43
40 cm SD	0.05	0.03	0.03	0.02	0.05
60 cm Mean	0.13	0.34	0.19	0.36	0.34
60 cm SD	0.03	0.01	0.03	0.04	0.03
100 cm Mean	0.11	0.27	0.27	0.37	0.22
100 cm SD	0.02	0.01	0.05	0.03	0.00
Total soil column Mean	0.18	0.32	0.34	0.30	0.37
Total soil column SD	0.07	0.05	0.10	0.06	0.10

The evergreen shrub was the only site to evidence clear propagation of dynamics in response to P throughout the soil column to 60 cm. Prior to leaf-out (Jan–early May), the larger deciduous site generally had 10 cm dynamics damped and mirrored in the lower two soil measurements, though not the intermittent layers, which was consistent with the presence of macropore flow.

There were also clear differences during the re-wetting period towards the end of the time series, with the evergreen conifer experiencing rapid re-wetting in all but the lowest layer, whereas the grassland site evidenced a more gradual, dynamic response in its upper three layers. Notably, the lowest grassland layer was extremely stable throughout the study period, indicated by the standard deviation (SD) in Table 2. Conversely, all layers of the sandier soiled evergreen shrub site re-wetted rapidly, whilst the larger deciduous site showed a more gradual rise in its upper layers, especially in contrast to the evergreen conifer and aforementioned evergreen shrub sites. Notably the smaller deciduous tree displayed, until the very end of the study period, the inverse of other sites in having lower soil layers being wetter than upper soils (Table 2 and Fig. 3). This was likely a result of the upper-most soils being siltier, whilst the increased disturbance (see ‘Vegetation plots’) would cause soils to behave fundamentally differently to their less disturbed counterparts.

#### Geophysical surveys

**Fig. 4:**
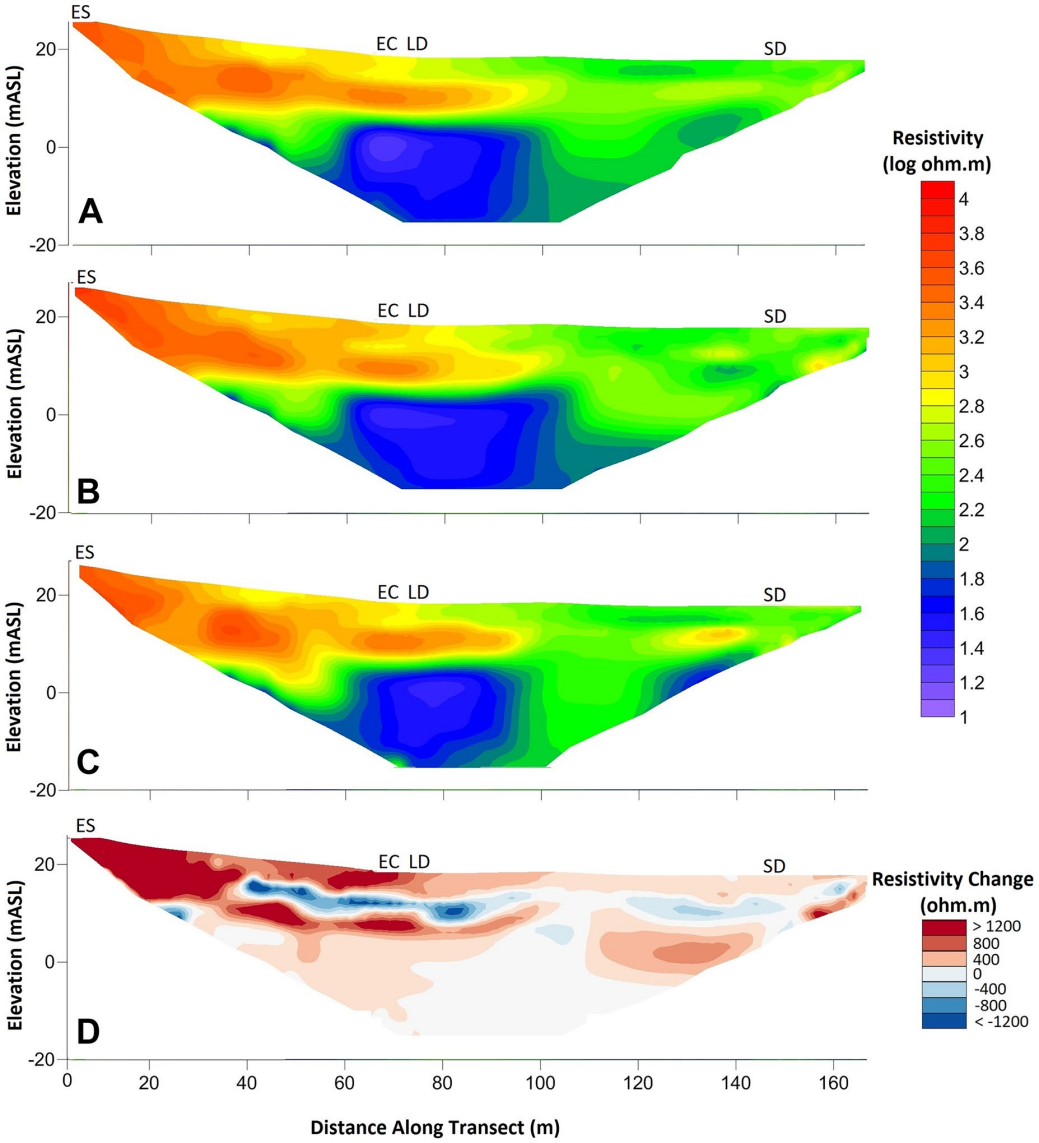
Geophysical survey results from October 2020 (**A**), July 2021 (**B**) and November 2021 (**C**). Panel **D** displays the difference in resistivity between July 2021 and October 2020. Panels **A**, **B** and **C** display results in natural logarithm of ohm metres, panel **D** in ohm metres. mASL; metres above sea level. The evergreen shrub (ES) was located at ~ 3 m along the transect, the evergreen conifer (EC) ~ 70 m, the larger deciduous (LD) ~ 75 m and the smaller deciduous (SD) ~ 147 m. In-print readers are directed to the online version. Figure created in Inkscape

The ERT surveys revealed that the upslope part of the transect (indicated by the western edge at ‘0 m’ in Fig. 1) had higher resistivities of around 1000 Ω-metres (ohm.m) or more (Fig. 4), reflecting glacial sandy material consistent with drier, sandier soils and lower soil water storage. This corroborated the soil column capacitance probes VWC values, particularly the drier conditions at the evergreen shrub plot (Table 2). At the point where the larger deciduous tree was located (~ 75 m along the transect; Fig. 4), the subsurface resistivity decreased, consistent with higher organic content and finer soil texture, and therefore higher soil water storage both within and below the soil probe profile. A layer of low resistivity of less than around 100 Ω.m at depth was further highlighted (panels A to C in Fig. 4), indicating the presence of glacial till or weathered metamorphic bedrock.

Seasonal time-lapse ERT surveys allowed us to indicate the relative seasonal changes in resistivity through computing the difference between two successive resistivity models which could then be used to infer changes in subsurface water storage. As would be expected, across the transect there was a clear drying trend between the wetter autumn (October 2020) and drier summer (July 2021) in the upper subsurface between approximately 0 and 5 m depth (panel D of Fig. 4), reflected by an increase in resistivity (by up to + 1000 Ω.m) between October 2020 and July 2021. This was most marked in the upslope section of the transect due to the sandier nature of the soils and steeper topography which encouraged more rapid drainage.

Further, from ~ 40 m distance a narrow band of about a few metres of decreased resistivity was observed (by up to 1000 Ω.m; Fig. 4D) which would indicate that this area was wetter than in October 2020, with the trend reversed between July and November of 2021 (not shown). This probably reflects a higher water table in July 2021 within the glacial sands given the preceding winters’ groundwater recharge. Conversely in November 2021, the water table would have been lower following summer dry conditions and before the upper soil moisture deficits were replenished in early winter which would subsequently percolate to groundwater recharge. Considering the year as a whole (Nov 2021–Oct 2020; not shown), there was a slight overall drying, which would be consistent with the lower than average rainfall during the study period.

#### Sapflux dynamics

**Fig. 5:**
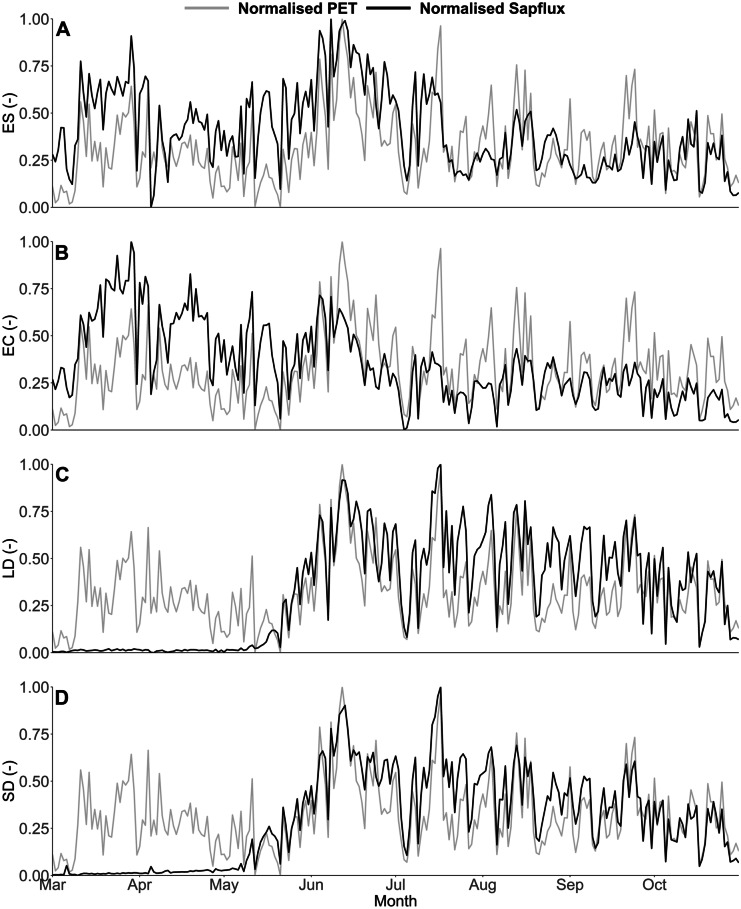
Normalised sapflux and normalised PET dynamics of the evergreen shrub (**A**), evergreen conifer (**B**), larger deciduous (**C**) and smaller deciduous tree (**D**). Figure created in RStudio

As expected, the deciduous trees recorded little sap movement until early May and the start of leaf-out facilitating transpiration (Fig. 5). Following this, normalised sapflux dynamics followed normalised PET dynamics very closely (normalisation was undertaken following the standard ‘min–max’ normalisation approach), indicating responsiveness to atmospheric moisture demands (Kuhlemann et al., 2021) and peaking in mid-July with net radiation (Fig. 3). Conversely, from the middle of May onwards, the evergreen conifer did not respond to atmospheric demand and, notably, experienced an early peak of sapflux rate at the end of March in response to an early spike in PET. A relative peak in evergreen shrub sapflux rate was also observed around the same period, whilst between July and October its sapflux rate was also mainly below the normalised PET (Fig. 5). However, during the soil re-wetting period of late September onwards, the evergreen shrub normalised sapflux rate, unlike the evergreen conifer, closely tracked PET, possibly indicating in the previous months the former may have been regulating soil moisture loss induced by the relative dryness of the soil (Kuhlemann et al., 2021).

#### Isotope data

P isotopes deviated somewhat from the expected seasonal sinusoidal pattern throughout the monitoring period (Fig. 3). In general, larger P events were associated with more depleted values, most notably in May, late September and October, though a depleted signal was also observed during a smaller event in early March. The most enriched signals occurred during a series of small P events in early April, whilst there was little variation observed between signals in late January to mid-February (Fig. 3).

**Fig. 6:**
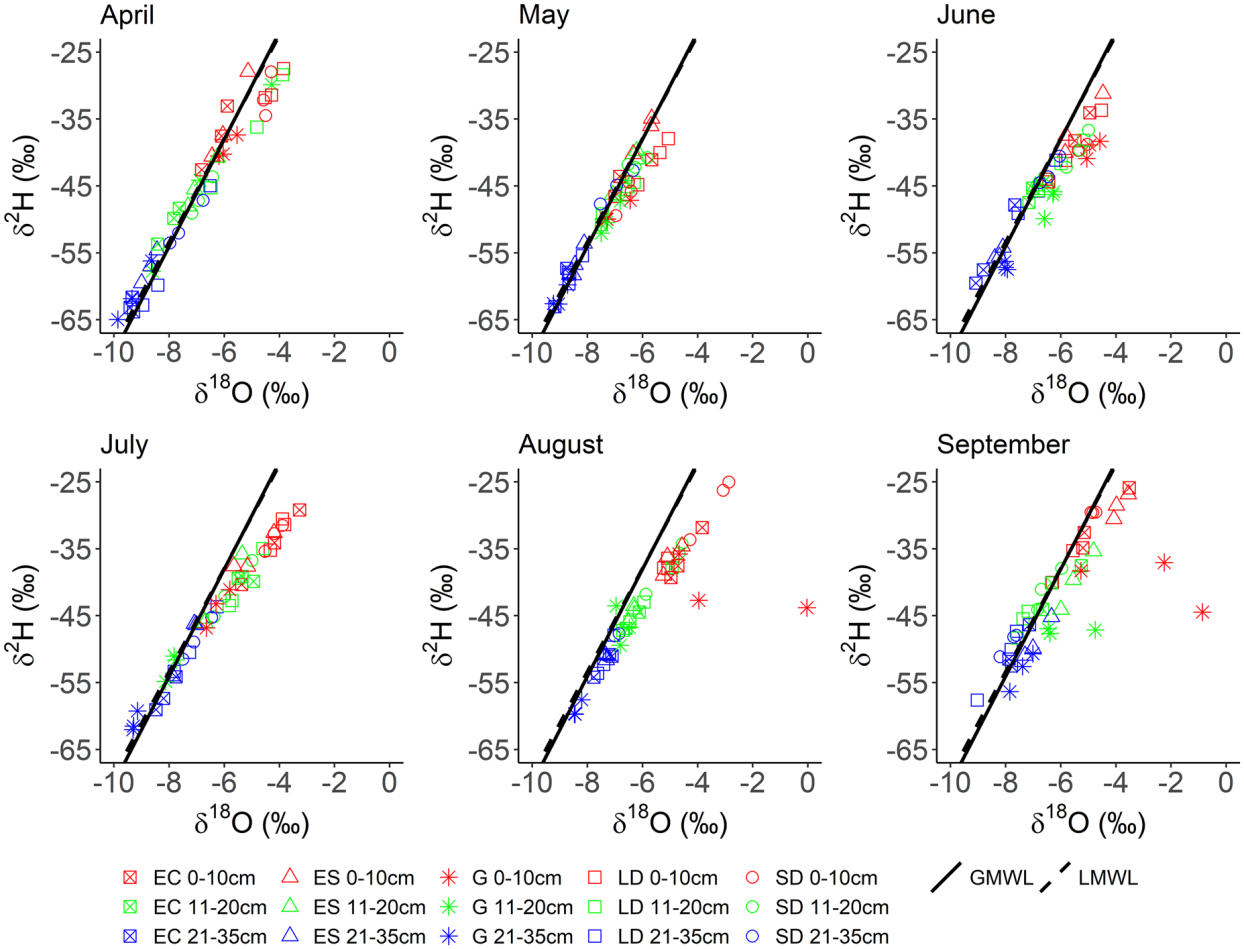
Soil water dual isotope plot timeseries from beneath the evergreen conifer (EC), evergreen shrub (ES), grassland (G), larger deciduous (LD) and smaller deciduous (SD) tree. In-print readers are directed to the online version. Figure created in RStudio

**Fig. 7:**
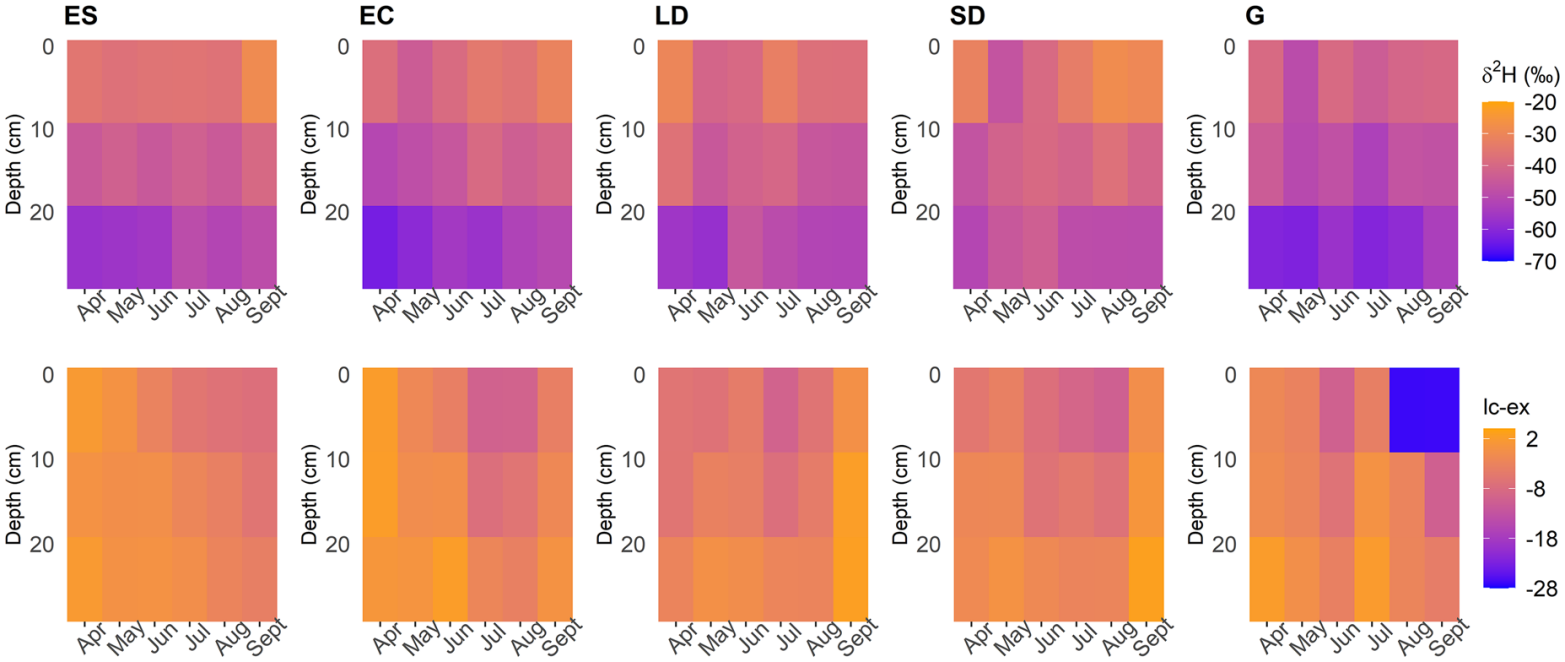
Isotopic composition (top row) and lc-excess (bottom row) timeseries of the different soil layers beneath the evergreen shrub (ES), evergreen conifer (EC), larger deciduous (LD), smaller deciduous (SD) and grassland (G) sites. Heat maps were derived from the average value of three replicates per vegetation type per month. In-print readers are directed to the online version. Figure created in RStudio

Generally, soil water isotopes became more depleted with depth, this being most evident at the grassland site and least so at the smaller deciduous site (Figs. 6 and 7). As a whole, between April and August soil isotopes under all vegetation types clearly showed the effects of evaporative fractionation down to 20 cm, with values plotting (Fig. 6) further below the global meteoric waterline (GMWL) and local meteoric waterline (LMWL—calculated from the P δ^2^H timeseries; slope = 7.72, intercept = 8.50). However, this evaporative signal was greatest at the grassland site, especially towards the end of the monitoring period. Furthermore, in contrast to the other sites, the calculation of lc-excess (as per Landwehr & Coplen, 2006) and dual isotope plotting showed that the deciduous plots had already experienced evaporation in April (Figs. 6 and 7), likely a result of increasing net radiation occurring prior to leaf-out. From ~ mid-June onwards, heat mapping further showed, in general, how pulses of fractionated water from the upper soil layers started to reach the lower depths at all sites, though most notably beneath the evergreen conifer and grassland. This trend of fractionated isotope signals started to generally reverse towards the end of the timeseries at all depths and sites as isotopic signals of increased precipitation events were reflected in soil isotope signals, with the notable exception of the grassland soils (Figs. 6 and 7).

#### Modelling ecohydrological fluxes

**Fig. 8:**
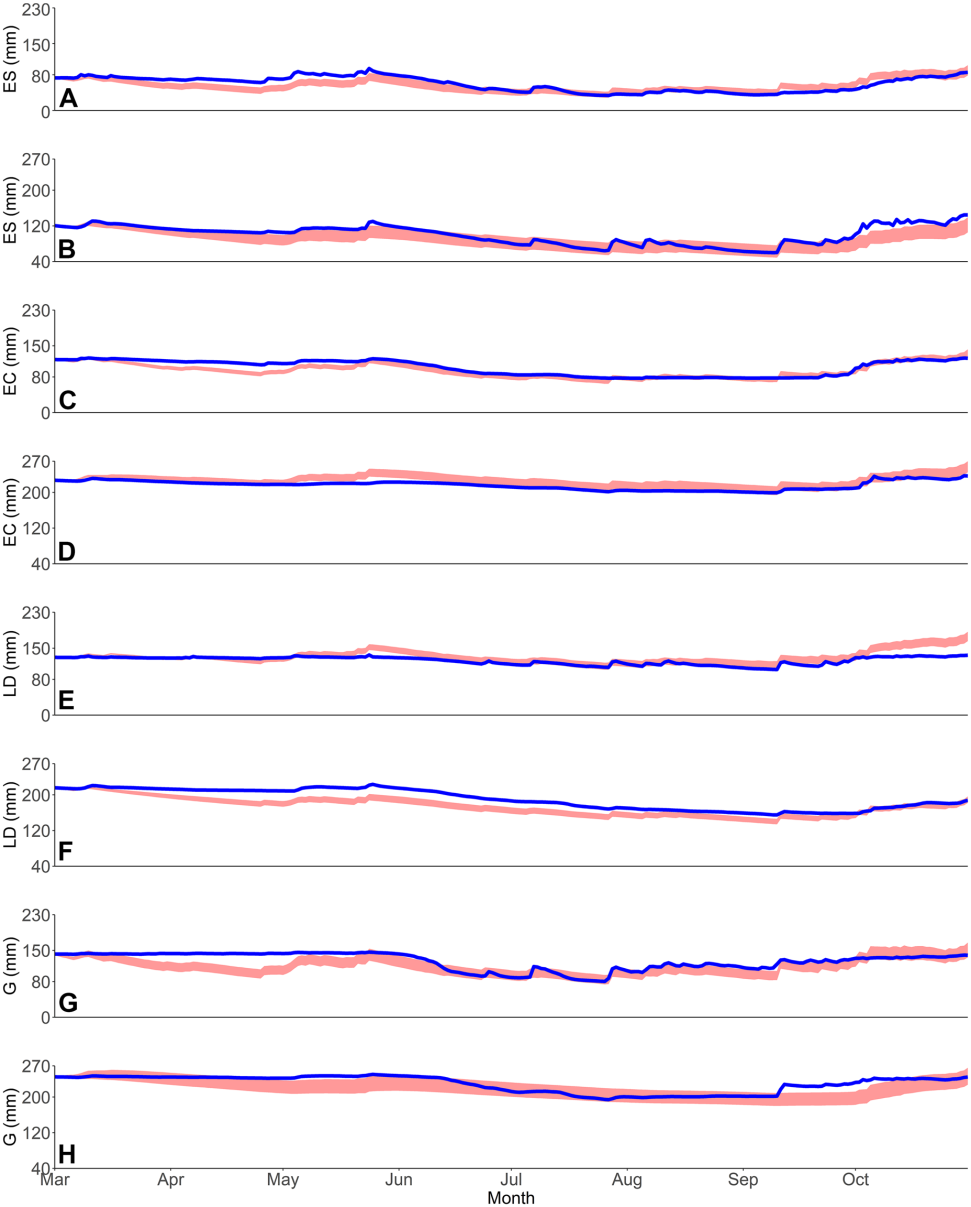
Observed (blue) and simulated (red band; 10th to 90th percentile of retained simulations) soil moisture for the evergreen shrub, evergreen conifer, larger deciduous and grassland upper soil compartments (**A**, **C**, **E** and **G**, respectively) and lower soil compartments (**B**, **D**, **F** and **H**, respectively). In-print readers are directed to the online version. Figure created in RStudio

The one-dimensional EcoHydroPlot modelling, calibrated against measured soil moisture, provided a reasonable and insightful first approximation of the ecohydrological fluxes from contrasting vegetation plots. From the calibration, 1039, 1221, 533 and 2295 parameter sets were retained for the evergreen shrub, evergreen conifer, larger deciduous and grassland applications, respectively. Simulations showed that the model was generally able to capture measured temporal soil moisture dynamics between heterogenous vegetation types (Fig. 8), with formal calibration metrics of these soil moisture simulations relatively consistent between sites (Table 3).

**Table 3:** KGE performance metrics of retained simulations

	Upper soil (10th percentile)	Upper soil (median)	Upper soil (90th percentile)	Lower soil (10th percentile)	Lower soil (median)	Lower soil (90th percentile)
*Evergreen shrub*	0.67	0.67	0.64	0.72	0.78	0.67
*Evergreen conifer*	0.86	0.88	0.87	0.83	0.88	0.83
*Larger deciduous*	0.41	0.43	0.40	0.84	0.87	0.87
*Grass*	0.69	0.70	0.69	0.72	0.78	0.75

**Fig. 9:**
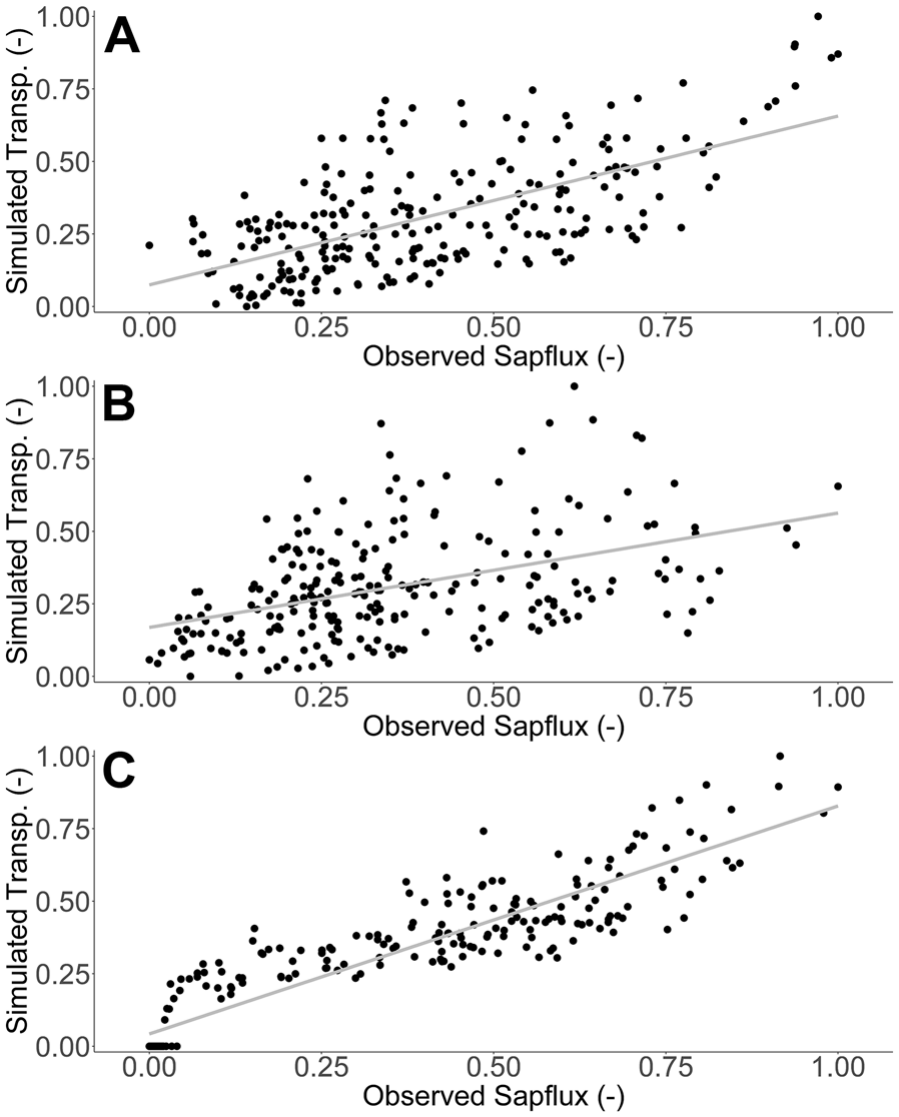
Regression plots between normalised observed sapflux data and normalised median (from retained simulations) transpiration data for the evergreen shrub (**A**; R^2^ = 0.40), evergreen conifer (**B**; R^2^ = 0.19) and larger deciduous (**C**; R^2^ = 0.84) applications. Figure created in RStudio

Furthermore, as an informal means of model evaluation, where daily normalised sapflux data was available, the relationship with median daily normalised transpiration estimates was significant (*P* < 0.05) and relatively strong in all but the evergreen conifer application, with R^2^ values of 0.40, 0.19 and 0.84 for the evergreen shrub, evergreen conifer and larger deciduous applications, respectively (Fig. 9). Moreover, calibration from common parameter sets resulted in marked differences in median parameter values for *S*_max_ (Table 1), which was a key parameter for scaling upper soil ecohydrological fluxes. The calibration, therefore, captured fundamental inter-site differences in soil moisture which were linked to changes in ecohydrological fluxes. For instance, transpiration rates in the evergreen shrub being suppressed when soil moisture decreased. However, overall the model did over-emphasise drying of soils during the initial time period, whilst it struggled to more accurately capture sometimes rapid re-wetting occurring towards the end of the period, especially in the upper soils of the larger deciduous area (Fig. 8). Furthermore, parameters such as *ks1* and *ks2* were not well constrained, whilst *rE* values were highly similar between vegetation types and poorly constrained from their initial range (Table 1). Despite this, confidence intervals were generally well constrained (Fig. 8), which increased confidence in the resulting ecohydrological flux estimates (Table 4).

**Table 4:** Median simulated ecohydrological flux values from retained simulations across the modelling period. 10th and 90th percentile bounds in parenthesis

	Evergreen shrub	Evergreen conifer	Larger deciduous	Grassland
Total evapotranspiration (mm)	214 (187,248)	265 (249,281)	224 (194,262)	193 (142,257)
Transpiration (mm)	155 (134,172)	205 (191,218)	128 (114,141)	113 (87,137)
Interception evaporation (mm)	55 (50,68)	56 (55,58)	57 (49,72)	51 (36,71)
Soil evaporation (mm)	4 (3,8)	4 (3,5)	39 (31,49)	29 (19,49)
Groundwater recharge (mm)	205 (174,238)	128 (110,148)	193 (171,217)	232 (201,267)
Tr/ET ratio	0.72 (0.72,0.69)	0.77 (0.77,0.78)	0.57 (0.59,0.54)	0.59 (0.61,0.53)
Tr/P	0.35 (0.30,0.39)	0.47 (0.43,0.50)	0.29 (0.26,0.32)	0.26 (0.20,0.31)

**Fig. 10:**
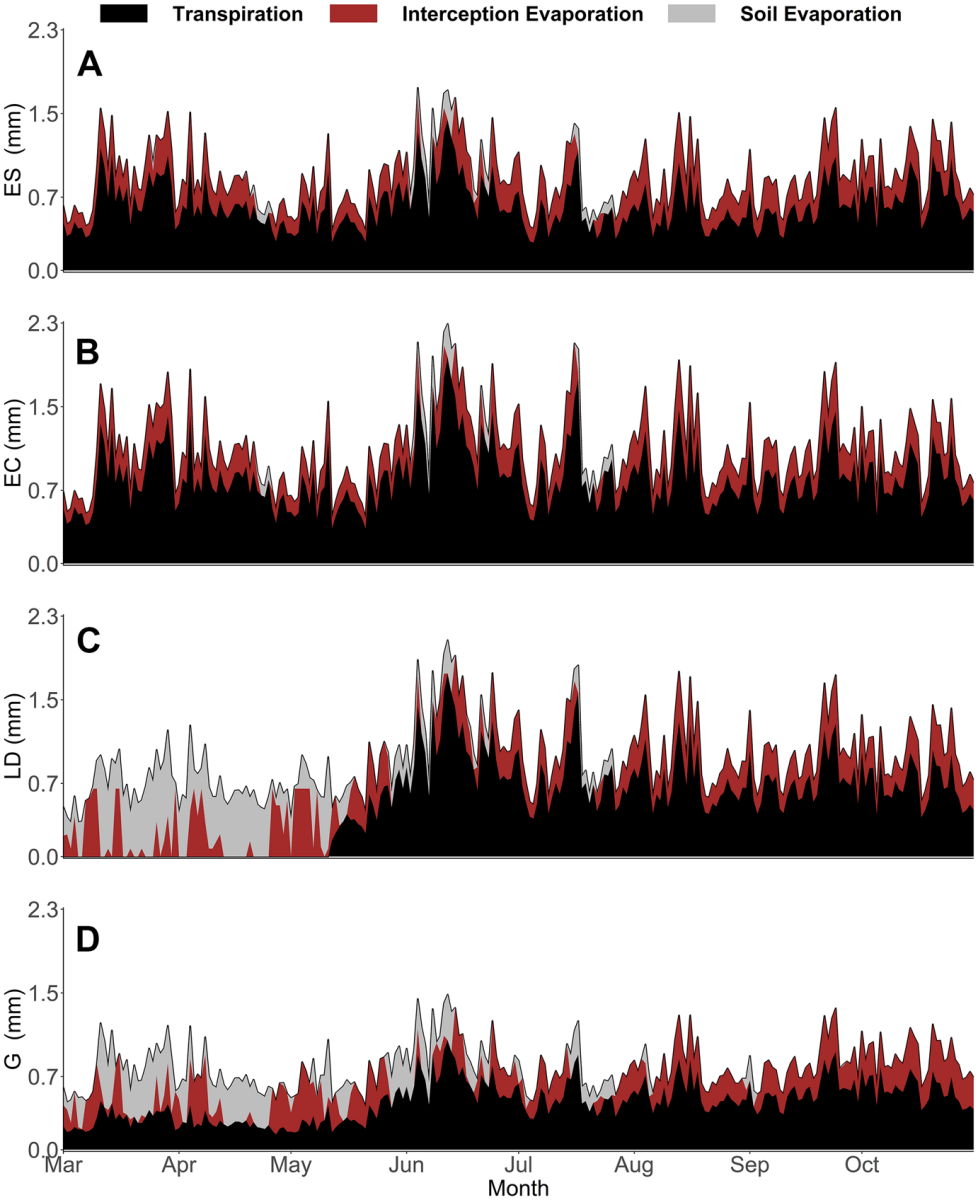
Timeseries of median (from retained simulations) transpiration, interception evaporation and soil evaporation estimated for the evergreen shrub (**A**), evergreen conifer (**B**), larger deciduous (**C**) and grassland (**D**) applications. In-print readers are directed to the online version. Figure created in RStudio

These estimates showed similarities between vegetation types, such as transpiration (Tr) being consistently simulated as the largest green water flux. Furthermore, Tr as a ratio to total evapotranspiration (Tr/ET; Table 4) within, though not between, evergreen and LAI variable groupings (e.g. larger deciduous and grassland for the latter) had clear similarities. Moreover, Int_E_ flux totals were relatively uniform between all four vegetation types (Table 4). However, there were substantial differences, such as a much lower Soil_E_ predicted under the evergreen species, whilst the larger deciduous site had the greatest total of Soil_E_, mainly occurring prior to leaf-out (Fig. 10). Further, Soil_E_ occurred under grassland during periods when it was not predicted elsewhere (e.g. September; Fig. 10), which is consistent with patterns seen in the observed soil isotope data (Figs. 6 and 7).

Tr dynamics also showed differences, with the evergreen conifer having nearly double the estimated Tr volume to the grassland and a third more than the evergreen shrub. The evergreen conifers increased rate of Tr was further reflected in the Tr/P ratio, with nearly half of all P volume being estimated as used for Tr (Table 4). All sites—apart from the larger deciduous tree—were predicted to have Tr occurring throughout the study period (Fig. 10), whereas the larger deciduous tree had no Tr until leaf-out. Despite this, the larger deciduous tree had total Tr nearing that of the evergreen shrub given that post leaf-out the tree had elevated levels of Tr (Fig. 10), as also implied by the sapflux data (Fig. 5). Overall, total evapotranspiration estimates increased in the order grassland < evergreen shrub < larger deciduous tree < evergreen conifer tree (Table 4).

Consequently, as a residual flux, *G*_r_ was also simulated as being variable under the different vegetation types (Tables 4 and 5). Considering the modelling period as whole, the grassland had the greatest total volume, with evergreen conifer having the least due to the simulations capturing well the relatively muted observed dynamics in the lower soil compartment (Fig. 8). The evergreen shrub had the second greatest *G*_r_, likely reflecting the freely draining sandy nature and upslope location (which may have promoted subsurface lateral flow at depth) of the soils—as suggested by geophysics results which showed substantial drying around the evergreen shrub location. *G*_r_ was generally higher during March and October, though the evergreen conifer displayed much reduced inter-month variation than other vegetation types (Table 5). Notably, *G*_r_ generally slowed substantially between March and April (Table 5), prior to the VMC of the soils markedly reducing around early June (Fig. 3), though following a reduction in precipitation frequency and intensity. This indicated the role of precipitation in propagating soil water displacement and encouraging *G*_r_ (whilst maintaining total VMC of the soil strata despite volume being lost via *G*_r_), though again this was lowest below the evergreen conifer. This precipitation-driven response was further emphasised between September and October when precipitation rates and intensity increased, helping to elevate rates of *G*_r_ (Table 5), despite the total VMC in the lowest layers remaining relatively unaltered (Fig. 8).

**Table 5:** Median simulated total monthly groundwater recharge (mm) per vegetation type. 10th and 90th percentile bounds in parenthesis

	**March**	**April**	**May**	**June**	**July**	**August**	**September**	**October**
*Evergreen shrub*	37 (30,47)	27 (23,32)	29 (25,33)	25 (22,29)	20 (17,22)	18 (16,21)	17 (15,20)	31 (27,35)
*Evergreen conifer*	18 (15,22)	15 (13,18)	19 (16,22)	17 (15,19)	14 (12,16)	13 (11,14)	12 (10,13)	20 (18,23)
*Larger deciduous*	37 (31,42)	27 (25,30)	29 (26,32)	24 (22,26)	19 (17,22)	18 (15,20)	16 (14,18)	24 (22,26)
*Grassland*	44 (35,56)	34 (29,39)	31 (28,34)	30 (27,32)	24 (21,27)	20 (17,23)	18 (15,21)	31 (28,35)

## Discussion

### Vegetation controls on the temporal dynamics of ecohydrological partitioning in UGS

We undertook hydrometeorological, soil moisture, sapflux and isotopic monitoring, along with the development and application of a one-dimensional plot-scale model, to determine the temporal and spatial dynamics of ecohydrological fluxes of an established, heterogeneous area of an UGS in northern Scotland. The study focussed on one growing season and captured general seasonal changes in hydrometeorological conditions, though P was 17% lower than average and, following an unusually warm March, April and May were cooler than average. Such variability may help place our results more firmly in the context of a changing climate and associated anomalies.

Soil moisture measurements, including stable water isotope monitoring, revealed how the temporal patterns of P differentially impacted soil storage dynamics beneath differing vegetation plots, similar to other work (c.f. Kuhlemann et al., 2021; Rahman et al., 2019; Wang et al., 2018; Zhang et al., 2019). Clearly, inter-plot differences in topography and soil type can be influential in such dynamics (Murphy et al., 2009), which was most obvious at and around the evergreen shrub, given the sandier soils and elevated topography. Consequently, more substantial drying and re-wetting dynamics were observed there in comparison to elsewhere on the study transect (Fig. 4), along with increased levels of simulated groundwater recharge. Moreover, results from the smaller deciduous site demonstrated how disturbance of UGS soils is likely influential in the stratification of volumetric water content (VMC) patterns, as lower soils were generally wetter than higher soil layers; the inverse of patterns was observed elsewhere (Table 2). Furthermore, at the beginning of the monitoring period, soil isotopes were less depleted at depth at the smaller deciduous site than elsewhere (Fig. 7). However, the precise impact of the role of disturbance in these observations is difficult to quantify, especially given the already siltier composition of the upper-most soils at the smaller deciduous site.

Such heterogeneities in topography and soil type are typical of UGS. Nonetheless, observations did evidence a clear impact of vegetation type on soil moisture VMC and stable water isotope signals, and so provided first indications of differences in ecohydrological partitioning. This was most apparent during the drier period between June and September; here, the grassland site saw a rapid and substantive drying of upper soils (and marked evaporative fractionation of the isotope signal) which were then responsive to P. This was in notable contrast to soils beneath the evergreen conifer that were less responsive post drying, as evidenced by soil moisture and isotope data (Figs. 3, 6 and 7). Such differences, given virtually identical soil types and topography, are most likely attributable to relative differences in canopy density which can influence soil moisture dynamics (Liu et al., 2016; Schume et al., 2003). At the grassland plot, the lower LAI permitted greater levels of P to reach upper soils during the summer months, and also enabled greater amounts of upper soil evaporation at peak PET demand; consequently, upper soil moisture levels were more dynamic, with the upper soil isotopic evaporative signal most notable beneath the grassland in comparison to other vegetation types (Figs. 6 and 7). Furthermore, the presence of grass in urban settings has previously been shown to increase soil infiltration (Wang et al., 2018) and thus, the drainage of upper soils during the drying period. Moreover, the thicker canopy of the evergreen conifer was likely influential in reducing the volume of *G*_r_ occurring during summer months in comparison to other sites (Table 5) given higher rates of interception and transpiration. Contrastingly to the grassland and evergreen conifer plots, soils under the two deciduous sites exhibited less marked drying and more damped responses to P, given the canopy LAI’s lay between those of grassland and evergreen conifer. These marked differences in drying trends between trees and grassland were not something observed in a previous similar, though geographically distinct, study (c.f. Figure 2 of Gillefalk et al., 2021). Our data also suggested more direct transfer of soil water between upper and lower soils under the larger deciduous tree at the start of the study period when distinct peaks in the 10 cm soil measurement were replicated, though damped, in the 60 cm layer, but not between. Such observations would be consistent with active macropore flow paths that can be created by rooting systems (Demand et al., 2019) which would be deeper and more extensive under larger trees.

Modelled green water fluxes from the soil store revealed, in contrast to findings by Gillefalk et al. (2021), that the larger deciduous site experienced greater levels of Soil_E_ than any other site, given the lack of canopy cover during the initial months—as was observed in the lc-excess of soil water isotopes during the time period (Fig. 7). However, once a canopy was fully established simulated Soil_E_ largely ceased (corroborated by changes in the isotope derived lc-excess values; Fig. 7), similar to the evergreen conifer and evergreen shrub (Fig. 10), though in contrast to the grassland. Furthermore, water losses via Tr were also markedly different between different vegetation types (though were consistently the largest green water flux as would be expected (Jasechko et al., 2013)), with the peak evergreen conifer sapflux rate occurring on 29th March (Fig. 5) and other vegetation types peaking during summer months. Such behaviour of the former is possibly a physiological artefact of the species being Korean and adapted to a snowmelt-driven soil moisture regime, which has been shown in other locations to control sapflux peaks (Cooper et al., 2020). However, on the same day, the evergreen shrub also experienced a relative high point in sapflux rates, indicating the increased atmospheric demand and their evergreen nature facilitated increased soil water uptake. Contrastingly, the deciduous tree could only transpire post leaf-out in May, though the tree then continually maximised sapflux in relation to PET (Fig. 5), similar to previous studies showing clear links between PET and daily Tr (c.f. Wang et al., 2019). These dynamics resulted in relatively similar total Tr volumes to the evergreen shrub (Table 4), though with a fundamentally different temporal pattern of soil water uptake (Fig. 10) given the evergreen shrub transpired at each timestep. With Tr/ET of 0.72, our estimated evergreen shrub ratio was found to be higher than a previously reported global mean of shrub Tr/ET (0.54; Gao et al., 2022; however, their study did not draw on data from the UK. Modelling estimates also implied that, due to a relatively low canopy density, the grassland experienced the lowest rate of Tr, accounting for 26% of P and 59% of ET (Table 4), the latter being similar to Zhang and Song’s (2021) grassland estimate of 50–70%. Given the low Tr and limited Soil_E_, the grassland had the lowest ET of all modelled habitats; contrasting to other studies (e.g. Gillefalk et al., 2021; Peters et al., 2011). Specifically, the latter study in Berlin identified evapotranspiration increasing in the order shrub < grassland < trees, whereas we found it to be grassland < evergreen shrub < trees (precisely, for the tree category, larger deciduous < evergreen conifer; Table 4). Reasoning for this difference is likely complex, though probably controlled by the species studied, especially given the mixed species nature of our grassland and differing definitions of ‘shrubs’, whilst geographical differences in soil type and hydroclimate may have further played a part.

As with previous research (c.f. Dawes et al., 2012; Gillefalk et al., 2021; Keese et al., 2005; Parizi et al., 2020), our modelling further indicated that vegetation type and characteristics strongly influence *G*_r_. These blue flux estimates were greatest beneath the grassland (232 mm; Table 4), despite the evergreen shrub plot having sandier, more freely draining soils (*G*_r_ = 205 mm; Table 4). Such a soil type may have been expected to facilitate *G*_r_ rates beyond those observed at other plots given soil hydraulics, and thus *G*_r_ rates, are contingent on soil type (Koeniger et al., 2016). However, the sandier evergreen shrub soils could hold lesser total volumes of water and therefore would have contributed less volume to *G*_r_ at each timestep (see ‘Model structure’). The evergreen conifer had the lowest *G*_r_, likely the result of the thicker canopy restricting inputs of P to upper soils, combined with high total Tr inhibiting percolation of soil water to depth. Indeed, this characteristic can be seen in the aggregated observed lower soil compartment data (Fig. 8) and corresponds to findings by Leuschner et al. (2022). Moreover, Kuhlemann et al. (2021) found higher interception and Tr under and by urban trees resulted in the slower turnover of soil water and older *G*_r_ in comparison to grassland and shrubs. Further, there have been clear differences in throughfall dynamics between broadleaved and coniferous trees (Levia et al., 2019). Such differences could impact *G*_r_ given how the effects of throughfall rate and dynamics could propagate through the soil profile and ultimately influence the rates and dynamics of *G*_r_, as indicated by results detailed in ‘Modelling ecohydrological fluxes’.

### Effectiveness of simple ecohydrological models in UGS

Our newly developed one-dimensional ecohydrological model (Fig. 2) yielded a valuable first approximation of ecohydrological fluxes in an UGS, using a data-driven approach. Importantly, and in contrast to other urban ecohydrological focussed models (c.f. Gillefalk et al., 2021; Meili et al., 2020), EcoHydroPlot has minimal parameterisation, does not require extensive datasets and is not computationally demanding, enabling it to be readily applied to four of the study plots. Furthermore, simulations showed the model was able to reproduce well the general soil moisture dynamics at all sites (Table 3) with differences in soil moisture levels and dynamics generally captured for different topographic positions, increasing confidence in other modelled outputs. Moreover, modelled flux dynamics were shown to be broadly consistent with sapflux data not used in the calibration process given the normalised relationship with modelled Tr was significant in all model applications (R^2^ values of 0.40, 0.19 and 0.84 for the evergreen shrub, evergreen conifer and larger deciduous application, respectively). Validation of a newly developed model via comparison to sapflux has been used previously (for example, Dye & Olbrich, 1993) and provides increased confidence in modelled flux dynamics, though this should be suitably circumspect in relation to the evergreen conifer given the comparatively low R^2^. Furthermore, soil isotope data broadly corroborated model simulations, by, for example, slightly increased levels of upper soil moisture fractionation being observed (as indicated by lc-excess heat mapping; Fig. 7) at the beginning of the study period beneath the larger deciduous site when the model also simulated elevated levels of Soil_E_.

From the simulations, we were able to ascertain, and have confidence in, the relative order of ecohydrological fluxes under differing habitats. For instance, evapotranspiration increasing in the order grassland < evergreen shrub < larger deciduous tree < evergreen conifer and groundwater recharge rates increasing in the order evergreen conifer < larger deciduous tree < evergreen shrub < grassland. Such information is vital for understanding heterogeneity in the ecohydrological functioning of UGS and consequently contributes to an evidence base to guide policy makers and land managers (Mathey et al., 2011) who wish to optimise the ecosystem service of an UGS, such as groundwater recharge or evapotranspiration to mitigate the urban heat island effect. However, there were limitations in the flux estimates derived from our relatively simple model, most notably the near uniform predictions of Int_E_ which were likely unrealistic given the variable canopy density and variation of observed P infiltration to the upper soil layers (Fig. 3). Furthermore, specific parameters such as *rE* were non-identifiable during calibration (Table 1) which may have led to wider uncertainty bounds. We also employed a relatively simple, though previously used (Šimůnek et al., 2013), approach to the splitting of PET into its component parts which could have impacted derived estimates in comparison to more complex, parametrised, approaches which would not have been suitable here. More complex models may also be able to take into account species age-derived differences in evapotranspiration and the subsequent governing equations, though in this study all species were well established limiting inter-species differences in age-related evapotranspiration. Moreover, our modelled results were derived without the inclusion of isotopic data in the calibration which could help differentiate soil evaporation and transpiration more accurately (Smith et al., 2021). Unfortunately, the collected soil isotope data was found to be of too low frequency for model incorporation due to insufficient information content for model calibration (though the isotope data was able to be used as an empirical derived ‘soft’ check on model simulations). Furthermore, if geophysical surveys were undertaken more frequently, the results may have been used as a further quantitative check on model simulations, specifically groundwater recharge, via the conversion of resistivities into a coarse VMC timeseries. Nonetheless, modelled results were on the whole broadly plausible in terms of overall quantitative flux estimates, consistent with observed data and yielded inter-plot ecohydrological flux order estimates similar to previous studies employing more complex modelling (Gillefalk et al., 2021). Therefore, in this application, our simpler one-dimensional model was successful in helping to understand the ecohydrological functioning of an UGS, suggesting it is a complementary approach to more complex, process-based models at sites where data may be limited.

### Wider implications

Our empirical and modelled results demonstrated clear differences in ecohydrological partitioning between vegetation types in an established UGS. Such results add to the growing body of evidence that the selection of vegetation type will directly impact water-related ecosystem services in UGS, especially supporting and regulating services (c.f. Rahman et al., 2015; Wang et al., 2018). For instance, in the energy-limited Scottish environment, we observed that soils beneath grassland maintained higher moisture levels during wetter periods (similar to Kuhlemann et al., 2021), experienced the most rapid drying and were most responsive to precipitation dynamics (Fig. 3). Consequently, the grassland plot had the greatest total volume of groundwater recharge (Tables 4 and 5) and thus this vegetation type may be more preferable than others studied when attempting to maximise this particular ecosystem service, as well as soil water storage more generally. Contrastingly, the grassland was, correspondingly, found to have the lowest total evapotranspiration and would therefore be less appropriate if green fluxes of water back to the atmosphere were desired, for example in areas wishing to mitigate the urban heat island effect.

Our results also demonstrated that both a larger deciduous tree and evergreen shrub (which was substantially smaller and younger) had similar rates of transpiration (Table 4). If generalisable, this has two important ramifications; firstly, evergreen shrub vegetation could be more easily incorporated into UGS given their smaller size and rapid establishment, whilst still yielding elevated levels of evapotranspiration. Secondly, given its evergreen nature, the evergreen shrub was shown to take up soil water for Tr at a relatively constant rate across the time period, whereas the larger deciduous tree transpired a similar amount but during a more limited time period when leaves were present (Fig. 10). These differences are important in providing environmental managers with a tool to focus time periods when water is transpired via the chosen vegetation type, and so contribute to potential ecosystem services such as urban cooling during periods when it is needed the most, such as spring and summer.

However, these results come with caveats given they are not only geographically but also site specific, whilst monitoring occurred over only 1 year and the modelling approach was relatively simple. Moreover, conclusions from point measurements, such as sapflux or soil moisture, in highly heterogenous environments need to be tentative. Nonetheless the coupling of empirical and modelling approaches has given a valuable first insight into the ecohydrological functioning of a heterogenous UGS in a previously unstudied area, whilst simpler model structures are successfully utilised in other areas of hydrological research (c.f., for example, Anshuman et al., 2021; Desclaux et al., 2018). Moreover, the results add to the weight of evidence (c.f. Gillefalk et al., 2021; Kuhlemann et al., 2021) which demonstrate UGS are ecohydrologically complex areas which, when managed correctly, can yield ecosystem services that may help to mitigate adverse hydrological impacts of urban expansion and climate change via, for example, increased rate of groundwater recharge. Finally, the study findings could contribute to improvements in typical urban drainage models given it is becoming more common to include green infrastructure in such models, though without, typically, distinguishing between vegetation types (e.g. Smith et al., 2023).

## Conclusion

We undertook ecohydrological monitoring in five soil-vegetation plots, along with the development and application of a one-dimensional ecohydrological model, in an established UGS to assess heterogeneities of inter-vegetation-related functioning over one growing season. Results showed clear differences in ecohydrological partitioning with evapotranspiration increasing in the order grassland < evergreen shrub < larger deciduous tree < evergreen conifer tree. Similarly, soils below grassland had greater soil water storage and groundwater recharge, but experienced more rapid drying, whilst the denser canopy of the evergreen conifer restricted summer P inputs to soil and restricted groundwater recharge. These results demonstrate that the ecohydrological functioning in heterogenous UGS is complex and the selection of specific vegetation types can directly impact the derived hydrological ecosystem services that might be required when employing green infrastructure to mitigate urban expansion and build resilience to climate change. In relation to the energy-limited environment of this Northern study site, results are most relatable in terms of identifying elevated levels of soil water storage and groundwater recharge beneath grassland, which could help mitigate the local effect of both climate change and urbanisation. Future work could usefully test if our simple data-driven modelling approach for understanding ecohydrological functioning is appropriate in differing habitats and geographic locations. Further, the inclusion of isotope data, and potentially geophysical survey data, for model calibration would improve confidence in process representation and facilitate water age estimations. Finally, more mechanistic, sub-daily dynamics could be investigated to understand the finer-scale functioning of urban ecosystems more fully.

## Data Availability

The datasets generated during and/or analysed during the current study are available from the corresponding author on reasonable request.
